# Special Issue on Mental Health and Well-Being in Adolescence: Environment and Behavior

**DOI:** 10.3390/ijerph18062975

**Published:** 2021-03-14

**Authors:** Javier Ortuño-Sierra, Beatriz Lucas-Molina, Félix Inchausti, Eduardo Fonseca-Pedrero

**Affiliations:** 1 Education Sciences Department, Faculty of Letters and Education, University of La Rioja, 26004 Logroño, Spain; felix.inchausti@unirioja.es (F.I.); Eduardo.fonseca@unirioja.es (E.F.-P.); 2 Developmental and Educational Psychology, Faculty of Psychology, University of Valencia, 46010 Valencia, Spain; beatriz.lucas@uv.es

Psychological problems in children and adolescent populations range from 10% to 20% [1]. Moreover, up to 75% of mental disorders seem to start before the age of 24 and the prodromic characteristics may have an earlier onset with a worse prognosis [1]. Furthermore, these symptoms may lead to more severe problems and mental health disorders with age [2]. For example, it is well known that depressed youngsters are at a higher risk of developing depression during adulthood, and that disruptive children and teenagers are more likely to have antisocial behavior as adults [1]. Therefore, and taking into account the short- and long-term consequences associated with psychological disorders, new efforts and resources are being devoted, with the aim of detecting and incorporating early prevention strategies and interventions [2].

It seems clear that an early identification of specific correlations between emotional and behavioral problems during childhood and adolescence before the onset of severe mental health problems could help with developing protective factors and preventing risk factors in order to mitigate, delay, or even prevent the onset of a clinical outcome. Promotion should always be the best strategy. Therefore, new studies analyzing psychological problems, as well as protective factors such as prosocial abilities, a sense of belonging to the community or the educational center, or self-esteem are still needed. For instance, new studies about phenotypically characterized samples of children and adolescents could help us to integrate different measures, including genetic, brain, psychometric, sociocultural, and neurobehavioral markers and may, potentially, allow us to articulate prophylactic and more effective interventions [3]. 

Adolescence is a developmental stage where different transformations at different levels occur. Among other things, adolescents have to cope with physical and psychological transformations and, in addition, establish their identity and their position in society. Moreover, it is a key period of human development for neurodevelopmental changes. The study of psychological and biobehavioral indicators related to emotional and psychological difficulties, well-being, and their connections with brain functioning could serve to help the understanding of developmental pathways of cognitive development related to subjective quality of life and mental health impairments [2].

This Special Issue of *IJERPH* on adolescent and well-being aims to incorporate different studies that could help us to further understand psychological and mental health issues in a relevant developmental stage, such as adolescence. Thus, the Special Issue includes papers that cover a wide range of aspects related to well-being and psychological issues, from methodological approaches to clinical interventions. We truly believe that the present Special Issue provides an insightful and thorough, up to date report on psychological well-being during adolescence from around the world that may help researchers and other stakeholders to increase their knowledge about a critical developmental stage such as adolescence.

For instance, Park, Kim, and Kim [4] analyzed the effects of sleep duration on suicide by gender. The study found that the effect of short sleep time on increasing suicidal ideation was 2.5 times higher in females when compared to males. This study could help us to prevent suicidal behavior during adolescence, which is, at this moment, the second highest cause of death at this developmental stage. In this regard, another study found that the prevalence of serious psychological distress and low self-rated mental health was 16.7% and 20.3%, respectively, with suicide ideation rates over 13%. A worrying 3.7% of adolescents have stated that they have attempted to commit suicide [5].

It is worth mentioning that the study conducted by Díez-Gómez et al. [6] identified four homogeneous subgroups for suicide behaviors: “low risk”, “suicidal act”, “suicidal ideation”, and “high risk for suicide”. This study is particularly relevant as it has a representative sample of more than 1500 adolescents. The four resulting subgroups revealed different patterns with regard to social–emotional adjustment. Those subgroups with the highest theoretical risk showed lower scores on subjective well-being and positive effects in addition to higher scores on emotional and behavioral problems and negative effects when compared to the non-risk subgroups. A relevant analysis about the specificities and similarities between suicidal youths and adults revealed that youths were somehow similar to adults, with comparable rates of personality disorders (especially borderline) and relapse and similar profiles of reasons for hospitalization in suicidal crises [7]. In this regard, new instruments analyzing suicidal behaviors are necessary, with the aim of preventing a problem such as suicide that has such a major impact on society [8]

Another study with Chinese students revealed that mental health issues were related to bullying, with a graded relationship between bullying victimization and mental health outcomes [9]. Bullying is a complex phenomenon that could have a big impact on mental health problems. In this regard, results from the study of Lucas-Molina et al. [10] could shed some light. They found that emotional regulation (ER) is an ability that precedes and predicts emotional knowledge (EK) in children. This idea may have relevant implications when establishing interventions with both children and adolescents suffering from and exerting bullying. Somehow related to this study, Malinakova et al. [11] revealed in a study that adolescence is a developmental period involving low differentiation between moral emotions like guilt and shame compared with adulthood. This is relevant in order to understand the different behaviors, attitudes, and profiles related to bullying [12]. Moreover, parenting styles could contribute to improving this kind of problem [13].

Mental health problems are a worldwide issue and more effort should be devoted, with the aim of detecting and preventing these kinds of problems early on [2]. The study of Luo et al. [14] with Chinese students revealed that the most frequent mental health problem was academic stress (58.9%). Moreover, they found that relevant variables, such as higher grades, physical disease, chronic constipation, alcohol consumption, engagement in sexual behavior, residence on campus, and living in nonurban areas and with single-parent families, were significantly associated with higher odds of having mental health problems. It is clear that mental problems have a clear impact on an individual’s wellbeing. A relevant study conducted in Spain revealed that variables such as students’ resilience and fear of failure, along with their sense of belonging, had a major impact on adolescents’ wellbeing [15]. In addition, promoting a positive school climate was found to be crucial to reducing bullying. Close to this topic, and unfortunately becoming more frequent nowadays, is intimate partner violence. The work of Ortuño-Sierra et al. [16] revealed that attitudes towards violence were more prevalent among men and that these attitudes were correlated with depressive symptoms. In addition, they confirmed the adequacy of an instrument for measuring this relevant construct that may allow one to prevent gender violence among adolescents.

In the prevention of mental health, it is also relevant to consider the context in which the person develops. The study of Escobar et al. [17] indicated that mental health issues were associated with hunger, distant relationships with parents, and family violence within the family context. In addition, the relationship between adolescents with a poorer family context and subjective wellbeing is well established [18]. In this regard, an insightful study provides a protocol-based program with an evidence-based intervention that was tailored specifically to the needs of runaway adolescents residing in youth shelters [19]. Considering the school context, feelings of loneliness and trouble sleeping were related to poor peer relationships and insecurity at school. A relevant aspect related to this could be the physical activity that children and adolescents practice. If the aim is to prevent mental health issues, instruments with adequate evidence of validity and reliability are necessary [20]. The study of Venetsanou et al. [21] provides the validation of a relevant instrument that allows for the measuring of physical activity. In addition, healthy habits can contribute to improving adolescent mental health and wellbeing. In this regard, another study analyzed the relationship between secondhand tobacco exposure and mental health issues [22]. It is necessary to understand that wellbeing during adolescence and in the transition to adulthood is a result of different relevant variables. For instance, the study of Morales-Rodríguez et al. [23] indicates that self-acceptance dimension, positive relationships, autonomy, environmental mastery, personal growth, and purpose in life played a key role in explaining the subjective wellbeing of undergraduate students.

To conclude, the present Special Issue encompasses different and relevant works related to subjective wellbeing, mental health, and related phenomena in children and adolescents. The studies in the Special Issue contribute valuable information that may help to understand the underlying etiology and course of psychological problems in relevant developmental stages. All of them may help to characterize the different developmental pathways that explain typical and non-typical development, with the aim of early detection and prevention of psychological issues that may transit to more severe problems with time.
